# Incidence and effects of Varicella Zoster Virus infection on academic activities of medical undergraduates - a five-year follow-up study from Sri Lanka

**DOI:** 10.1186/1471-2334-10-117

**Published:** 2010-05-14

**Authors:** Suneth B Agampodi, Samath D Dharmaratne, Vasanthi Thevanesam, Sameera Dassanayake, Prabhashini Kumarihamy, Ashani Ratnayake

**Affiliations:** 1 Department of Community Medicine, Faculty of Medicine and Allied Sciences, Rajarata University of Sri Lanka, Sri Lanka; 2 Department of Community Medicine, Faculty of Medicine, University of Peradeniya, Sri Lanka; 3 Department of Microbiology, Faculty of Medicine, University of Peradeniya, Sri Lanka

## Abstract

**Background:**

The adult population in Sri Lanka is having high level of susceptibility for Varicella Zoster Virus (VZV) infection. Among medical undergraduates, 47% are VZV seronegative. The purpose of the present study was to determine the incidence of VZV infection in medical undergraduates in Sri Lanka, and to describe the effects of VZV infection on their academic activities.

**Methods:**

A retrospective cohort of medical undergraduates' susceptible for VZV infection was selected from the University of Peradeniya, Sri Lanka. Data on the incidence of VZV infection (Chickenpox) during their undergraduate period was collected using a self-administered structured questionnaire. A second questionnaire was administered to collect data on the details of VZV infection and the impact of it on their academic activities. VZV incidence rate was calculated as the number of infections per 1,000 person years of exposure. Descriptive statistics were used to describe the impact of VZV infection on academic activities.

**Results:**

Out of the 172 susceptible cohort, 153 medical undergraduates were followed up. 47 students reported VZV infection during the follow up period and 43 of them participated in the study. The cumulative incidence of VZV infection during the period of five and half years of medical training was 30.7%. Incidence density of VZV infection among medical undergraduates in this cohort was 65.1 per 1,000 person years of follow-up. A total of 377 working days were lost by 43 students due to the VZV infection, averaging 8.8 days per undergraduate. Total academic losses for the study cohort were; 205 lectures, 17 practicals, 13 dissection sessions, 11 tutorials, 124 days of clinical training and 107 days of professorial clinical appointments. According to their perception they lost 1,927 study hours due to the illness (Median 50 hours per undergraduate).

**Conclusions:**

The incidence of VZV infection among Sri Lankan medical undergraduates is very high and the impact of this infection on academic activities causes severe disruption of their undergraduate life. VZV immunization for susceptible new entrant medical undergraduates is recommended.

## Background

Sero-epidemiological reports[1-3] and reports from the Infectious Disease Hospital[4] in Sri Lanka demonstrate strong evidence of a high level of susceptibility for Varicella Zoster Virus (VZV) infection among the adult population in Sri Lanka. According to the Indoor Morbidity and Mortality Report (IMMR) in 2003, 1,749 patients with VZV infection were admitted to government hospitals in Sri Lanka, out of which 15 were fatal[5]. Disease outbreaks have been frequently reported from institutional settings, but data are not available on these outbreaks. Even though these reports are suggestive of high susceptibility, accurate morbidity and mortality data on VZV infection is scarce in Sri Lanka. In 2006, VZV infection was made a notifiable disease in Sri Lanka[6] and the Epidemiology Unit launched a passive surveillance system in order to provide baseline data on VZV morbidity. In 2007, the total number of notifications received by the Epidemiology Unit was 3,435[7]. This number increased by 59.5% by 2008, with 5,493 patients contacting VZV infection[8]. In a tropical country with a population of 20 million, this is an under estimate of the actual incidence of VZV infection. Further, it does not provide data on high-risk groups. The true incidence rate, which is of utmost importance in determining high-risk groups and generating vaccination policies is yet to be described for Sri Lanka.

In 1995, the United States of America introduced the VZV vaccine to their National Immunization Program [9] which was followed by several other countries in the western world. The epidemiology of VZV infection in these countries had changed drastically with the introduction of the vaccine[10]. In most of the countries in the South Asian region including Sri Lanka, the VZV vaccine is not included in the National Immunization Programs at present. In Sri Lanka, it is available in pro-profit private sector providers. Even in the private sector, the use of the VZV vaccine was very low according to a study done in Colombo, Sri Lanka. They reported that only 4.2% of children less than three years were immunized with VZV vaccine[11]. At present, the immunity to the disease is acquired by contracting the natural infection in a large proportion of the population.

Health care workers have long been identified as a high risk group for VZV infection [12]. Most of the developed countries have well defined vaccine policies for healthcare workers and institutional policies for undergraduates in health care settings [13-17]. Susceptibility of medical undergraduates to common infectious diseases has been studied widely and findings of these studies provide strong evidence to support these vaccination policies [18-21]. In 2008, it was reported that the susceptible proportion of medical undergraduates for VZV infection was as high as 47% in Sri Lanka [1]. Nevertheless, further data are required to make policy decisions regarding the disease incidence and the impact of VZV infection among medical undergraduates on their academic activities. The present study was conducted to determine the incidence of VZV infection in medical undergraduates in Sri Lanka, and to describe the effects of VZV infection on their academic activities.

## Methods

In the years 2002 and 2003, Faculty of Medicine, University of Peradeniya, Sri Lanka conducted a sero-epidemiological study to estimate the susceptibility of new-entrant university undergraduates to VZV infection. Undergraduates entering the Faculties of Medicine and Engineering during the period of 2002 and 2003 were invited to participate in this study. Findings of this sero-epidemiological study and the methodology was published elsewhere [1]. We used the susceptibility data of medical undergraduates of this sero-epidemiological study for the present study.

The present study was conducted as a retrospective cohort study. A cohort of medical undergraduates' susceptible for VZV infection were selected from the previous study [1] using the following methodology. First, all medical undergraduates with a positive history of VZV infection were excluded from the study sample, and the remaining medical undergraduates underwent a VZV antibody test (anti-VZV antibody IgM ELISA kit-Human Diagnostics, Germany). All the VZV antibody negative medical undergraduates were selected as the susceptible cohort (study sample) for the present study.

The susceptible (VZV antibody negative) cohort of medical undergraduates was contacted after their final examination to collect data for the present study. Data on the incidence of VZV infection during their undergraduate period was collected using a self-administered structured questionnaire. Self reported history of VZV infection was used as a valid measure of VZV infection in this study. For those who reported a history of VZV infection during the study period, a second self administered structured questionnaire was used to collect data on the details of their VZV infection and to measure the impact of it on their academic activities. The month and the year of acquiring the infection was used to calculate the period at risk in months for each participant. All who acquired the disease were removed subsequently from the at risk population to calculate the incidence density of VZV infection. Details on the duration of staying away from studies, effect of the infection on their academic activities and impact on examinations were obtained from this questionnaire.

Data was analysed using SPSS 13 statistical software. Incidence rate was calculated as the number of infections per 1,000 person years of exposure. Total person years of exposure was calculated using the exact period at risk. Once a student is infected, he/she is not at risk anymore and time spent as undergraduate after the infection was not included in the denominator. In this calculation, each student who has not had VZV during the undergraduate period contributed by 5.5 years, whereas students who had VZV contributed less. Descriptive statistics were used to describe the impact of VZV infection on academic activities. Ethical clearance for the study was obtained from the Ethical Review Committee, Faculty of Medicine, University of Peradeniya, Sri Lanka.

## Results

**Figure 1:**
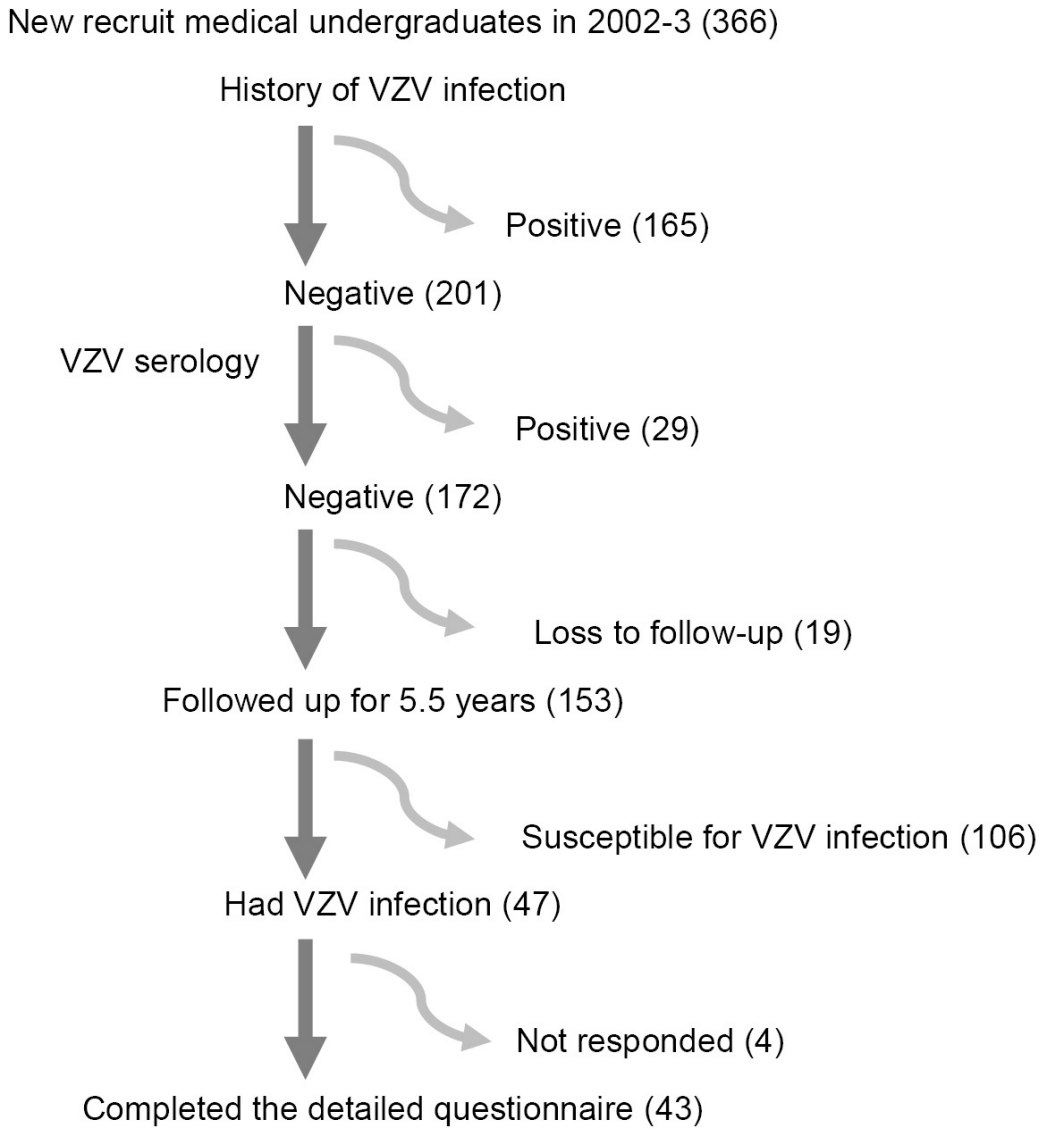
**Study flow chart with number loss to follow-up in each step**.

Figure 1 shows the study flow chart with the number of medical undergraduates followed-up in each step of the study. The initial susceptible study cohort consisted of 172 (89 males and 83 females) undergraduates from two batches of entrants entering the Faculty of Medicine, University of Peradeniya, Sri Lanka in 2002 and 2003. We could only follow-up 153 susceptible and the proportion lost to follow up was 11.0%.

**Figure 2:**
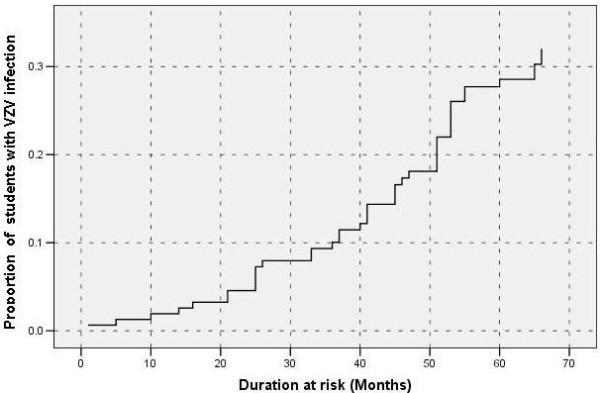
**Cumulative proportion of medical undergraduates with VZV infection during the period of 66 months (five and half years of undergraduate period)**.

During a period of five and a half years, 47 medical undergraduates had VZV infection, which accounted for 30.7% cumulative incidence of VZV infection during their undergraduate period. The total person years of exposure were 722. Incidence density of VZV infection among medical undergraduates in this cohort was 65.1 per 1,000 person years of follow-up. Figure 2 illustrate the cumulative incidence of VZV infection calculated in this study.

Of the 47 medical undergraduates who had VZV infection, 43 completed the second questionnaire (response rate - 91.5%). Table 1 shows their demographic profile.

**Table 1:** Characteristics of the 43 medical undergraduates who had VZV infection.

**Mean age (SD) years**	**26.7**	**(0.95)**
**Sex**		
Male (%)	29	(67.4)
Female (%)	14	(32.6)
**Ethnicity**		
Sinhalese (%)	42	(97.7)
Tamil (%)	01	(2.3)

The age range of the VZV infected undergraduates was 25 to 28 years. The majority (67.4%0 were males and belonged to the Sinhalese ethnic group (97.7%). Out of the 43 medical undergraduates who responded, only three (7.0%) had moderate to severe complications; two had pneumonia and the other had a widespread secondary bacterial infection. The median duration of fever was three days with an inter-quartile range of 1-3 days. The majority, (88.4%) were residing outside their homes (in a university hostel or a boarding place) when they developed the symptomatic disease. Five (11.6%) medical undergraduates acquired the infection while they were at home.

Only two (4.6%) were hospitalized during the illness, and both had VZV pneumonia. All but one went home for the total duration of their illness. Nine (20.9%) went home on the second or third day of diagnosis and 27 (62.8%) went home on the same day of diagnosis.

Table 2 shows the academic activities lost due to the VZV infection. A total of 377 working days were lost by them, averaging 8.8 days per medical undergraduate. Total academic losses for the study cohort were; 205 lectures, 17 practical sessions, 13 dissection sessions, 11 tutorials, 124 days of clinical appointments (clinical training in hospitals in third and fourth years) and 107 days of professorial appointments (final year clinical training in Medicine, Surgery, Gynaecology/Obstetrics and Paediatric wards). According to their individual perception, they lost 1,927 study hours due to the illness. This accounted for a median of 50 hours (IQ range 28-60) per medical undergraduate.

**Table 2:** Perceived effect of VZV infection on academic activities among the 43 medical undergraduates who developed the disease during the 66 months of follow-up.

Effect of VZV infection on academic activities	N	%
No effect on academic activities	6	13.9
Reduced study duration	20	46.5
Repeated a clinical appointment (more than 1 week)	5	11.6
Could not sit an examination	4	9.3
Reduced performance in examinations (passed exams without classes)	4	9.3
Failed one or more subject	2	4.7
Delayed internship	2	4.7

Impact of the disease on academic performance and undergraduate activities is showed in Table 2. Twenty (46.5%) medical undergraduates thought the disease reduced their study duration considerably, but did not affect their performance at examinations. Four (9.3%) medical undergraduates thought that due to the study hours lost during VZV infection they could not perform well at the examination and obtained a general pass without honours. Another four were prevented from sitting an examination (other than the final examination) due to the infection and two (4.7%) claimed that they did not pass because of the infection. Two medical undergraduates (4.7%) could not sit the final examination, which delayed their internship by six months. Three students who had complications reported similar loss of academic activities and two of them thought that it affected their examination performance.

## Discussion

This study confirmed that medical undergraduates are a high-risk population for VZV infection, reporting an incidence density of 65.1 per 1,000 person years of follow-up. According to population based studies and surveillance reports from other countries, the incidence of VZV infection among adults is higher in tropical countries compared to temperate countries [22]. Nevertheless, the incidence of more than 50 per 1,000 person years of follow-up is much higher than the rates reported in the pre-vaccine era in several countries [23-25]. These reported incidence data do not indicate true incidence, because the denominator in most of these estimations includes the immune population, not the susceptible population, which can severely underestimate the incidence. The reported incidence for medical undergraduates in Sri Lanka is very high compared to these other studies [23-25] even when the difference in the denominator is taken into account.

This high incidence density is not surprising in the study population of the present study due to the high exposure rate among them. Preventing outbreaks in such a population is impossible without a proper immunization policy. The present study shows that at the time of the diagnosis, 88% of the medical undergraduates were residing at hostels. Even though most of them went home on the same day of the diagnosis (27/38, 62.8%), by the time they were diagnosed, they had enough time to infect others in these crowded hostels. Most of these hostel rooms in the study setting are occupied by around 4 undergraduates although they are designed for two. In addition during lectures, practical sessions, dissection sessions, tutorials and clinical appointments, the close contact with a large number of others facilitates the effective transmission of infection.

The present study further highlights that only two medical undergraduates with complications were admitted to a health care facility (hospital) for treatment of the infection. If the others were self diagnosed or diagnosed by informal examinations by a faculty member, these infections may not have been reported to the communicable disease notification system of the country. Therefore, the actual disease burden estimations based on routine notification data will not include medical undergraduates as a high risk group. If this situation also prevails in the general population, the reported data will be a severe underestimate of the actual disease burden in Sri Lanka.

Susceptible medical undergraduates will start work as medical officers after the completion of their degree. Only around 30% of the study cohort was infected during the follow-up period and more males were infected while the majority of the females remained susceptible. VZV infection during adult life and especially during pregnancy has been shown to entail severe complications on the mother and the child [26]. Therefore, there is an increased risk to these female medical officers who are in their reproductive age from VZV infection once they start work.

Further, our study shows that less than 5% of the susceptible students acquired the infection during the first 20 months where pre-clinical subjects are taught. A very high percentage (>25%) of students were infected during the period where they engaged in clinical activities. Some of the students may have worked in wards where there were pregnant mothers, elderly patients and immunocompromised patients. These patients were at high risk of acquiring VZV with complications as a hospital acquired infection.

The effect of VZV infection on academic activities, which have not been documented previously in Sri Lanka, shows a huge impact on the life of the individual medical undergraduate. These factors are difficult to measure in cost effective terms. Yet, it is evident that immunization programs can have a remarkable effect in their future, especially for those who could not perform to their optimum due to the VZV infection.

Currently, most developed countries have immunization policies for medical undergraduates [13,15]. The present Sri Lankan health policy does not include immunization against VZV infection and only two vaccines (Hepatitis B and Rubella) are given in an ad hoc manner in some of the universities. Either the National Advisory Committee on Infectious Diseases or individual universities should make their decision on an immunization policy. We recommended including a VZV immunization policy for medical undergraduates in Sri Lankan medical schools.

### Limitations

This study has several limitations where the interpretation of results should be done within these limitations. First, VZV infection was confirmed by recall history of medical undergraduates, but not by serological evaluation. Recall bias could have affected the study in several ways. VZV could cause subclinical infections or very mild clinical disease, where the classical clinical picture is not apparent. This mild or subclinical infections which was not diagnosed or was not remembered by the respondent could underestimate the true incidence of VZV in this study population. Secondly, respondents may have misdiagnosed a rash with fever due to other causes as a mild case of VZV infection, resulting in an overestimation of VZV incidence. The study was conducted after the final year exams and the recall period was 5.5 years. Even though they could recall major impacts such as failing in exams, number of lectures/practicals missed could be affected by the recall bias. To minimise the overestimation, we inquired about the minimum number of classes missed.

## Conclusions

The incidence of VZV infection among medical undergraduates is very high and the impact of this infection on academic activities causes severe disruption of an undergraduate's life which could affect his/her future. The authors strongly recommend that University health authorities consider VZV immunization for susceptible new entrant medical undergraduates/university students in Sri Lanka.

## Competing interests

The authors declare that they have no competing interests.

## Authors' contributions

ASB participated in the design, performed the data analysis and interpretation and prepared the manuscript. SDD participated in the design and in the coordination of the study and helped in data interpretation and manuscript preparation. VT conceptualized the study and participated in design and data interpretation. DDGBMS, KKWMPP, and RRMASK participated in the data collection, data analysis and manuscript preparation. All authors read and approved the final manuscript.

## Pre-publication history

The pre-publication history for this paper can be accessed here:

http://www.biomedcentral.com/1471-2334/10/117/prepub
